# Synergistic Effect of Dietary Betaines on SIRT1-Mediated Apoptosis in Human Oral Squamous Cell Carcinoma Cal 27

**DOI:** 10.3390/cancers12092468

**Published:** 2020-08-31

**Authors:** Nunzia D’Onofrio, Luigi Mele, Elisa Martino, Angela Salzano, Brunella Restucci, Domenico Cautela, Marco Tatullo, Maria Luisa Balestrieri, Giuseppe Campanile

**Affiliations:** 1 Department of Precision Medicine, University of Campania Luigi Vanvitelli, Via L. De Crecchio 7, 80138 Naples, Italy; nunzia.donofrio@unicampania.it (N.D.); elisa.martino@unicampania.it (E.M.); 2 Department of Experimental Medicine, University of Campania Luigi Vanvitelli, Via Luciano Armanni 5, 80138 Naples, Italy; luigi.mele@unicampania.it; 3 Department of Veterinary Medicine and Animal Production, University of Naples Federico II, Via F. Delpino 1, 80137 Naples, Italy; angela.salzano@unina.it (A.S.); brunella.restucci@unina.it (B.R.); giuseppe.campanile@unina.it (G.C.); 4 Experimental Station for the Industry of the Essential Oils and Citrus Products (SSEA), Special Agency of the Chamber of Commerce in Reggio Calabria, Via G. Tommasini 2, 89125 Reggio Calabria, Italy; dcautela@ssea.it; 5 Marrelli Health—Tecnologica Research Institute, Biomedical Section, Via E. Fermi, 88900 Crotone, Italy; marco.tatullo@tecnologicasrl.com

**Keywords:** δ-valerobetaine, betaine, oxidative stress, apoptosis, SIRT1, oral carcinoma

## Abstract

**Simple Summary:**

Betaines are important human nutrients widely distributed in plants, animals, and dietary sources. δ-valerobetaine (δVB) is a naturally occurring betaine with antioxidant, anti-inflammatory and anticancer activities. The aim of our study was to investigate the possible synergism between δVB and the structurally related γ-butyrobetaine (γBB) by testing the in vitro anticancer activity in head and neck squamous cell carcinomas. Combined δVB and γBB caused a marked inhibition of cell proliferation and induction of apoptosis in Cal 27 cells. The increased reactive oxygen species accumulation influenced the nuclear expression of SIRT1. Gene silencing with small interfering RNA confirmed the role of SIRT1 in the apoptotic cell death. Synergism of δVB and γBB is useful for novel strategies to optimize their content in meat, milk and dairy products to sustain human health and wellbeing.

**Abstract:**

Betaines are food components widely distributed in plants, animals, microorganisms, and dietary sources. Among betaines, δ-valerobetaine (*N,N,N*-trimethyl-5-aminovaleric acid, δVB) shares a metabolic pathway common to γ-butyrobetaine (γBB). The biological properties of δVB are particularly attractive, as it possesses antioxidant, anti-inflammatory and anticancer activities. Here, we investigated the possible synergism between δVB and the structurally related γBB, to date unexplored, by testing the in vitro anticancer activity in head and neck squamous cell carcinoma cell lines, FaDu, UM-SCC-17A and Cal 27. Among cell lines tested, results indicated that betaines showed the highest effect in reducing Cal 27 cell proliferation up to 72 h (*p* < 0.01). This effect was enhanced when betaines were administered in combination (δVB plus γBB) (*p* < 0.001). Inhibition of cell growth by δVB plus γBB involved reactive oxygen species (ROS) accumulation, upregulation of sirtuin 1 (SIRT1), and apoptosis (*p* < 0.001). SIRT1 gene silencing by small interfering RNA decreased the apoptotic effect of δVB plus γBB by modulating downstream procaspase-3 and cyclin B1 (*p* < 0.05). These findings might have important implications for novel prevention strategies for tongue squamous cell carcinoma by targeting SIRT1 with naturally occurring betaines.

## 1. Introduction

Betaines are quaternary ammonium compounds originating from amino and imino acids widely distributed in plants, animals, microorganisms, and dietary sources [1,2]. A growing body of evidence shows that betaines are important human nutrients that participate in the methionine cycle acting as methyl donors. Compounds belonging to this class, such as glycine betaine, stachydrine, ergothioneine, γ-butyrobetaine (γBB), and δ-valerobetaine (δVB) show protective effects against cardiovascular disease and cancer [3,4,5,6,7,8].

Among betaines, δVB and γBB are particularly abundant in ruminant meat, milk, and dairy products [9,10,11]. δVB (*N,N,N*-trimethyl-5-aminovaleric acid) is produced by rumen microbiota from the transformation of N^ε^-trimethyllysine and is likely degraded by the intestinal host microbiota via γ-butyrobetaine hydroxylase enzyme, thus suggesting a possible metabolic connection between δVB and γBB [9,10]. In vitro biological activities of δVB include inhibition of β-oxidation of fatty acids in mice cardiomyocytes, anti-inflammatory effects in endothelia damaged by hyperglycemia, and cytotoxic effects in human colon adenocarcinoma cells [6,8,12]. In vivo and clinical evidence indicate that δVB inversely correlates with metabolic syndrome markers after the intake of whole grain-rich diets [13,14]. The molecular mechanism through which δVB triggers colon cancer cell autophagy and apoptosis involves altered cellular redox homeostasis and sirtuin 6 (SIRT6) modulation [8].

Another member of seven mammalian homologs of Sir2, sirtuin 1 (SIRT1), also plays a crucial role in stress response and chemoresistance. Studies on the role of SIRT1 as a tumor suppressor or oncogene are controversial and may depend on the tumor type [15]. Under massive levels of DNA damage and loss of tumor suppressors or checkpoints, SIRT1 overexpression promotes cancer formation [15,16,17]. In oral cancer, SIRT1 expression correlated with tumor repression and its downregulation at transcriptional level is linked to malignant transformation, invasion and metastasis [18,19,20,21]. Moreover, in vitro studies on Cal 27 cells and xenograft mouse model support the potential of SIRT1 as tumor suppressor in oral cancer and provide the rationale for the use SIRT1 activators from dietary source in the setting of new prevention strategies [22].

A deeper knowledge of the biological basis of the antitumor effects of δVB is tempting, especially in light of the possible mechanism of action of δVB occurring through the reduction of l-carnitine uptake by inhibiting the organic l-carnitine cation transporter type 2 [12], known to have an altered expression in several cancer cell lines. In particular, this mechanism may take part to an alternative to glucose source of energy for cancer cells growth and proliferation by supplying l-carnitine for β-oxidation [23,24]. The present study aimed at evaluating the possible anticancer effects of milk betaines in oral squamous cell carcinomas [25]. In particular, in light of recent evidence showing that pure δVB displays more pronounced health-promoting properties when in combination with other milk components [6,8], particular emphasis was placed on the evaluation of its possible synergistic/additive effects with γBB, given the remarkable structural resemblance of these two betaines.

## 2. Results

### 2.1. Effect of Milk Betaines on Cell Viability, Proliferation and Sirtuins

**Figure 1:**
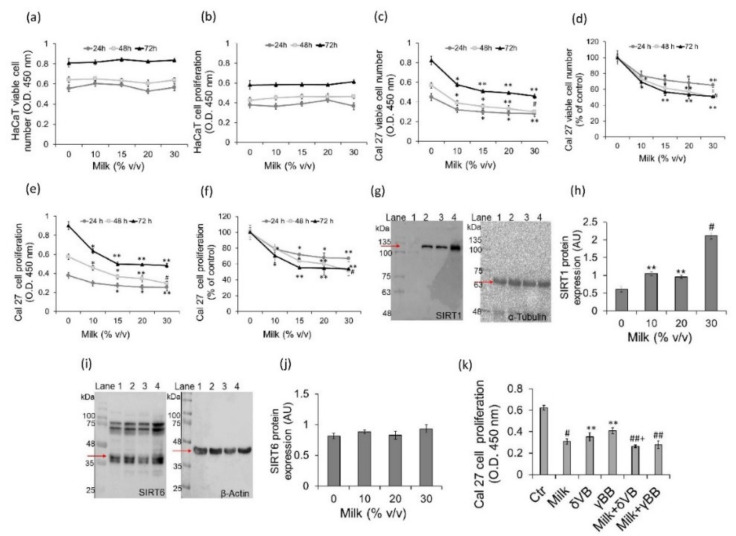
Effect of milk on cell viability, proliferation and sirtuins. Milk was centrifuged at 3000× *g* for 15 min at 4 °C to remove fat globules. Skimmed milk was then filtered through a 5 μm Millipore filter followed by filtration through an Amicon Ultra 0.5 mL centrifugal filter with a 3-kDa molecular weight cut-off. Before being used, milk extracts were filtered through 0.22 μm Millipore filters. Enrichment of milk was performed by adding 2 mM δVB or 2 mM γBB (**a**–**f**) Cells were treated with increasing volumes of milk (up to 30% *v*/*v*) for 0, 24, 48, and 72 h. Control cells were grown in medium containing the same volume (% *v*/*v*) of HBSS-10 mM Hepes. Cells viability and proliferation were assessed by Cell Counting Kit-8. The optical density (OD) values at time 0 h did not differ among treatments with milk (from 0 up to 30% *v*/*v*); mean values were (**a**) 0.483 ± 0.0097 (**b**) 0.298 ± 0.017, (**c**) 0.263 ± 0.019 (**e**) 0.217 ± 0.021. (**g**,**h**) SIRT1 and (**i**,**j**) SIRT6 protein expression levels in Cal 27 cells after 48 h of treatment with milk. Lane 1 = Ctr, lane 2, milk 10% *v*/*v*, lane 3 = milk 20% *v*/*v*, lane 4 = milk 30% *v*/*v*. (**k**) Cal 27 cell proliferation performed after 48 h of treatment with milk (30% *v*/*v*) supplemented with 2 mM δVB or 2 mM γBB. Results are expressed as arbitrary units (AU) with * *p* < 0.05 vs. Ctr, ** *p* < 0.01 vs. Ctr, ^#^ *p* < 0.001 vs. Ctr, ^##^ *p* < 0.0001 vs. Ctr, ^+^ *p* < 0.05 vs. milk.

Cell proliferation and viability were firstly evaluated by testing milk, an aliment of fundamental importance for humans due to the remarkable content of substances with high nutritional and functional values, including betaines. Noteworthy, recent data indicate that 3-kDa milk extracts show higher efficiency in inhibiting colon cancer cell viability compared to betaine alone [8,9,10]. Milk extracts were tested on FaDu, UM-SCC-17A, and Cal 27 cells. Immortalized human keratinocytes HaCaT cells were used as a control non-tumor cell line. Treatments with 3-kDa milk extracts for 48 h did not affect HaCaT cell viability and proliferation (Figure 1a,b). On the contrary, same treatments induced dose-dependent cytotoxic and antiproliferative effects on Cal 27 cells with the highest potency at 30% (*v*/*v*), (*p* < 0.001 vs. Ctr) (Figure 1c–f), corresponding to 32.4 µmol/L of δVB and 9.61 µmol/L of γBB [10]. Optical density (OD) values at time 0 h did not differ among treatments with milk extract (from 0 up to 30% *v*/*v*). As for FaDu, a lower effect on cell viability was observed compared to Cal 27 cells (Supplementary Figure S1). Effects on cell viability were even to a less extent in UM-SCC-17A cells (Supplementary Figure S1). Milk treatments also induced a dose-dependent increase of SIRT1 protein expression with the highest effect at 30% (*v*/*v*) (*p* < 0.001 vs. Ctr) (Figure 1g,h). SIRT6 protein expression was not affected by milk treatments (Figure 1i,j).

In order to investigate the biological component mainly responsible for the antiproliferative activity of milk, similarly to previous studies [8], cells were treated with milk enriched with 2 mM δVB (milk + δVB) or 2 mM γBB (milk + γBB). Results indicated that milk + δVB showed the higher antiproliferative activity compared to milk alone (*p* < 0.05 vs. milk), whereas milk + γBB showed a positive trend in the reduction of Cal 27 cell proliferation compared to milk (Figure 1k).

### 2.2. Effects of Pure δVB and γBB on Cancer Cell Proliferation

**Figure 2:**
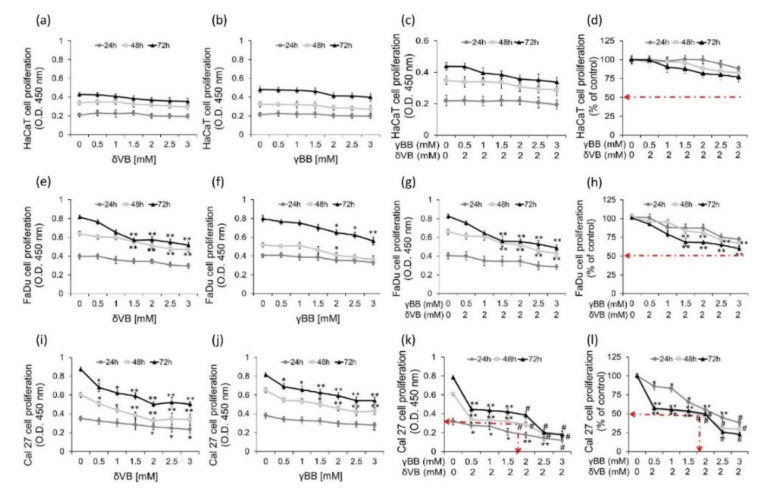
Inhibition of cell proliferation. Cell proliferation assays after exposure to different concentrations of δVB or γBB (up to 3 mM) or to δVB (2 mM) *plus* serial concentrations of γBB (0.5, 1, 1.5, 2, 2.5, 3 mM) cells for different times (24, 48 and 72 h) were performed in (**a**–**d**) HaCaT (**e**–**h**) FaDu and (**i**–**l**) Cal 27. The IC_50_ in Cal 27 cells was determined at 48 h incubation with 2 mM δVB *plus* 1.62 mM γBB. Control cells were grown in medium containing the same volume of HBSS-10 mM Hepes. Cell proliferation inhibition was assessed using Cell Counting Kit-8 assay. Values represent the mean±SD of four independent experiments. * *p* < 0.05 vs. Ctr, ** *p* < 0.01 vs. Ctr, ^#^ *p* < 0.001 vs. Ctr.

To investigate the possible additive or synergistic effect of δVB and γBB, we next evaluated HaCaT, UM-SCC-17A, FaDu, and Cal 27 cell proliferation after exposure to pure single or combined betaines (2 mM δVB plus serial concentrations of γBB). Results indicated that single and combined betaines, even at the highest concentration of γBB (3 mM), did not show any cytotoxic effect on HaCaT cells (Figure 2a–d). In contrast, δVB and γBB showed a time- and dose-dependent capacity in inhibiting FaDu and Cal 27 cell proliferation. As for FaDu cells, the highest inhibition was reached at 72 h with 3 mM δVB (36.4%) and 3 mM γBB (30.1%), without reaching the IC_50_ (Figure 2e–h). UM-SCC-17A cell proliferation was only weakly affected at 72 h treatment with γBB and δVB, both at the highest concentration of 3 mM (*p* < 0.05 vs. vehicle) (Supplementary Figure S2). Among cancer cell lines, the cytotoxicity induced by betaines resulted more pronounced in Cal 27 cells, with a high efficiency at 48 h of treatment with 2 mM δVB and 2.5 mM γBB (45% and 35% of cell proliferation inhibition, respectively) (*p* < 0.01 vs. vehicle) and extended up to 72 h (Figure 2i–l). Cal 27 cells responded to the combined treatment with betaines reaching the IC_50_ at 2 mM δVB plus 1.62 mM γBB (*p* < 0.001 vs. Ctr). The resulting combination index (CI) was equal to 0.99112, indicating a synergistic effect (Supplementary Figure S2). Based on these results, further studies aimed at elucidating cellular events and molecular targets were performed by using single δVB (2 mM) and γBB (2.5 mM) or combined δVB and γBB (δVB + γBB) (2 mM + 1.62 mM).

### 2.3. Cell Cycle Phase Modulation

**Figure 3:**
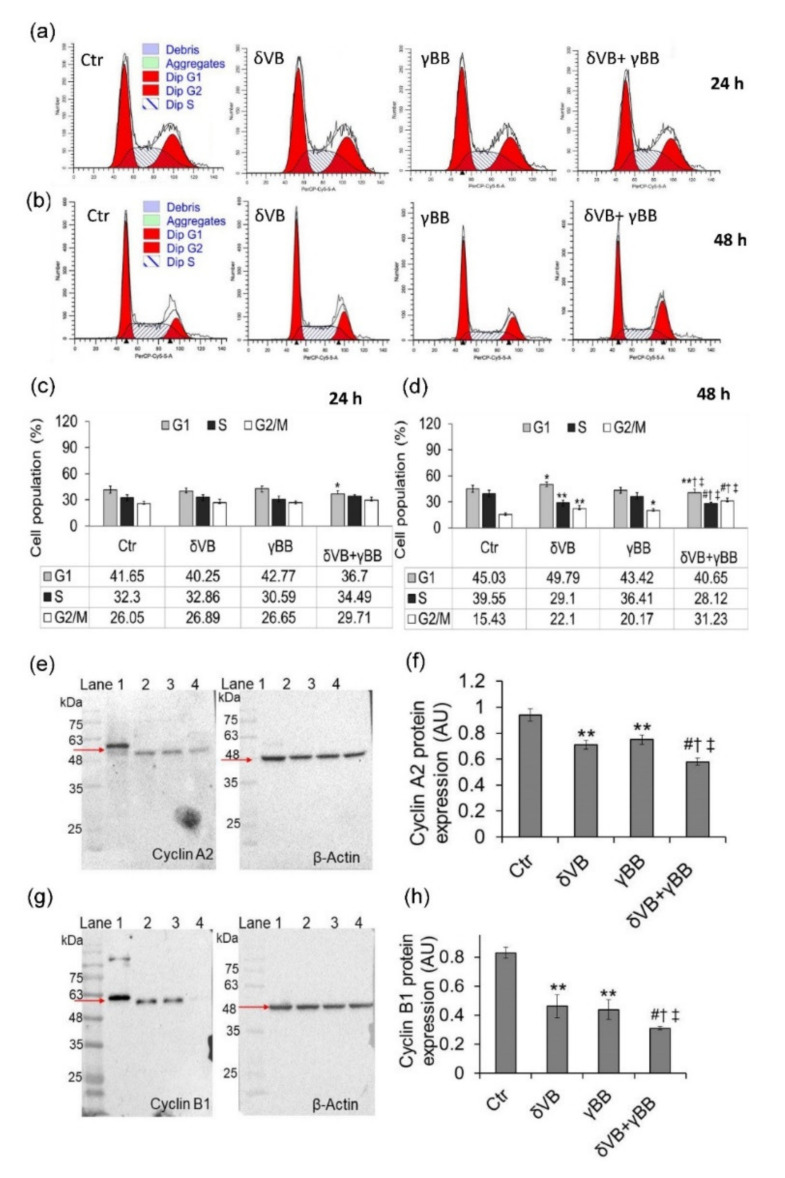
Cell cycle analysis. (**a**–**d**). Representative cell cycle analysis and average of Cal 27 cell cycle distribution. Cells were treated with vehicle (Ctr), δVB (2 mM), γBB (2.5 mM), or δVB +γBB (2 mM + 1.62 mM) for 24 h (**a**,**c**) and 48 h (**b**,**d**). Cell cycle distribution was assessed by flow cytometry collecting PI fluorescence as FL3-A (linear scale) and analysis by ModFIT software (Verity Software House, Becton Dickinson, Topsham, ME, USA). For each sample at least 10,000 events were analyzed. (**e**–**h**) Representative full-length blots of Western blotting analysis of cyclin A and cyclin B1 in cells treated for 48 h. Lane 1 = Ctr; lane 2 = δVB, lane 3 = γBB, lane 4 = δVB + γBB. Protein expression was determined after normalization with internal control (β-actin) with Image J software and quantified using β-actin. Values are expressed as arbitrary units (AU). * *p* < 0.05 vs. Ctr, ** *p* < 0.01 vs. Ctr, *^#^ p* < 0.001 vs. Ctr, ^†^ *p* < 0.05 vs. δVB, ^‡^ *p* < 0.05 vs. γBB.

Cell cycle analysis showed an effect of δVB + γBB on FaDu cells only at 72 h of treatment with an accumulation of cells in G2/M and G1 phase and a decrease of S phase (*p* < 0.01 vs. Ctr) (Supplementary Figure S3). In Cal 27 cells, the effects of combined betaines (δVB + γBB) on cell cycle emerged as early as 24 h of exposure but was more consistent at 48 h (Figure 3). At 24 h of exposure, a depletion of cells in the G1 phase was observed only in the presence of δVB + γBB (*p* < 0.05 vs. Ctr) (Figure 3a,c), whereas at 48 h of exposure cells responded to treatments with both single and combined betaines (Figure 3b,d). In details, δVB treatment determined an increase of cell percentage in G2/M phase (*p* < 0.01 vs. Ctr) and a decrease in S phase (*p* < 0.01 vs. Ctr). γBB induced only an increase of cell percentage in G2/M phase (*p* < 0.05 vs. Ctr). Of note, exposure to δVB + γBB determined a decrease of the cell percentage in the G1 and S phases (*p* < 0.001 vs. Ctr, *p* < 0.05 vs. δVB, *p* < 0.05 vs. γBB) and a more consistent increase of cells in G2/M phase compared to control cells (about 2-fold) (*p* < 0.001 vs. Ctr) and cells treated with single betaines (about 1.5-fold) (*p* < 0.05). The synergistic effect of betaines at 48 h was also reflected by the highest potency in downregulating cyclin B1 and cyclin A expression levels compared to single betaines (*p* < 0.05) (Figure 3e–h).

### 2.4. Apoptotic Cell Death

**Figure 4:**
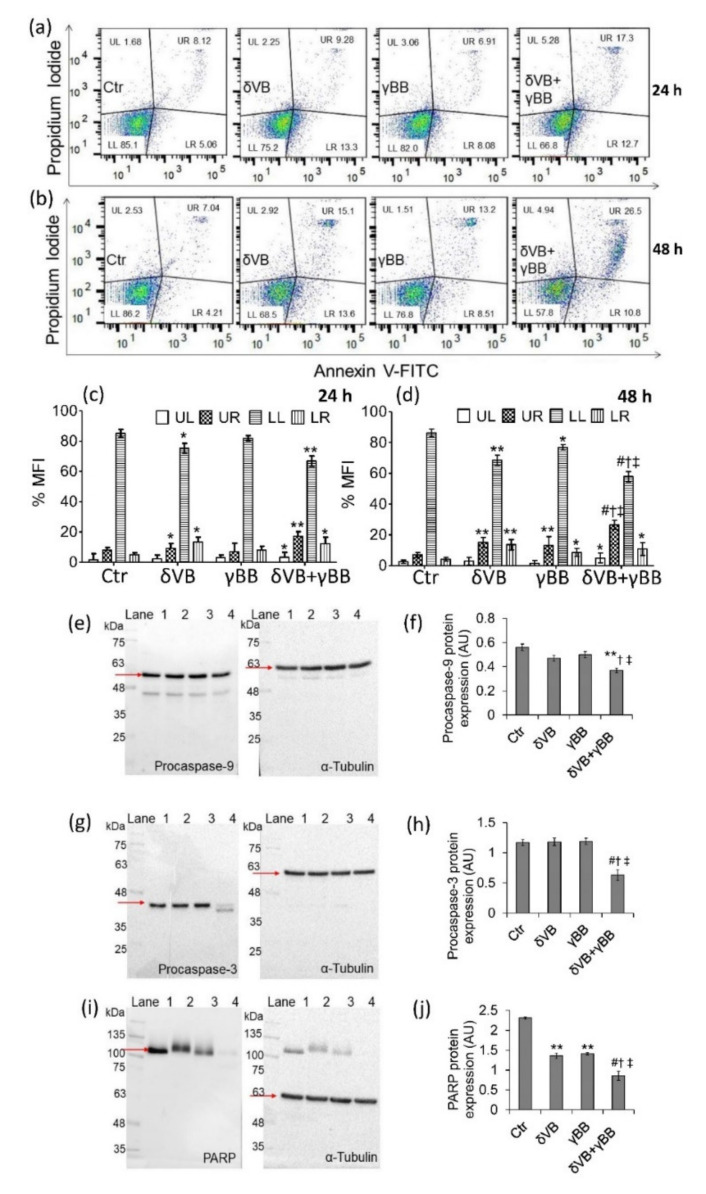
Apoptotic Cal 27 cell death. (**a**–**d**). Representative dot plots and analysis of annexin V-FITC and PI-stained cells analyzed by flow cytometry. The percentage of cells is reported in the quadrants; lower left (LL), viable cells; upper left (UL), necrotic cells; lower right (LR), early apoptotic cells; upper right (UR), late apoptotic cells. Data are expressed as mean ± SD of *n* = 3 experiments. At least 10,000 events were acquired. Full-length blots and protein expression levels of (**e**,**f**) procaspase-9, (**g**,**h**) procaspase-3, and (**i**,**j**) PARP from treated for 48 h with δVB (2 mM), γBB (2.5 mM), δVB +γBB (2 mM + 1.62 mM), or HBSS-10 mM Hepes (Ctr). Lane 1 = Ctr; lane 2 = δVB, lane 3 = γBB, lane 4 = δVB + γBB. Analysis of densitometric intensity was calculated with Image J software. Arbitrary units (AU) of protein expression were quantified using α-tubulin, as internal control. * *p* < 0.05 vs. Ctr, ** *p* < 0.01 vs. Ctr, *^#^ p* < 0.001 vs. Ctr, ^†^ *p* < 0.05 vs. δVB, ^‡^ *p* < 0.05 vs. γBB.

The mechanism of Cal 27 cell death was evaluated by measurements of annexin V/PI expression. Results indicated that at 24 h treatments, δVB alone decreased the number of live cells (*p* < 0.05 vs. Ctr) and increased late (*p* < 0.05 vs. Ctr) and early apoptotic (*p* < 0.05 vs. Ctr) cell number, whereas γBB showed no effects. The combined treatment with δVB + γBB was more efficient in decreasing the number of live cells (*p* < 0.01 vs. Ctr) and increasing the number of cells in late apoptosis (*p* < 0.01 vs. Ctr) (Figure 4a,b). At 48 h of treatments, a more consistent number of annexin V/PI positive cells was observed following exposure to δVB + γBB (*p* < 0.05 vs. δVB, *p* < 0.05 vs. γBB, *p* < 0.001 vs. Ctr), supporting the synergism between betaines (Figure 4c,d). Conversely, FaDu cell apoptosis was occurred only following treatment with the δVB + γBB for 72 h (*p* < 0.05 vs. Ctr) (Supplementary Figure S4). Although suspended cells were not removed during sample preparation, a lack of cells in subG1 was detected. However, betaines determined a significant increase of necrotic cells that do not undergo DNA fragmentation (Figure 4c,d), in line with recent evidence in colon cancer cells where only minimal percentage of cells in subG1 was observed [8]. Evaluation of procaspase-3 and procaspase-9, after 48 h of treatment with δVB + γBB showed a downregulation of protein expression levels (*p* < 0.01 vs. Ctr, *p* < 0.05 vs. δVB, *p* < 0.05 vs. γBB) (Figure 4e–h). An increased expression of caspase-3 protein at a lower molecular weight, ascribable to caspase-3 cleaved form, was observed in cells exposed to δVB + γBB (*p* < 0.001 vs. Ctr, *p* < 0.05 vs. δVB, *p* < 0.05 vs. γBB) (Figure 4e–h). A decrease in the expression of poly (ADP-ribose) polymerase (PARP) was observed both after treatment with single betaines (*p* < 0.01 vs. Ctr) and with combined betaines (δVB + γBB) (*p* < 0.001 vs. Ctr, *p* < 0.05 vs. δVB, *p* < 0.05 vs. γBB) (Figure 4i,j). Although the expression of caspase-9 and PARP cleavage products were not appreciable, the decrease of their respective full-length along with the annexin V/PI positive cells strongly supports the activation of apoptosis.

### 2.5. SIRT1 Activation

**Figure 5:**
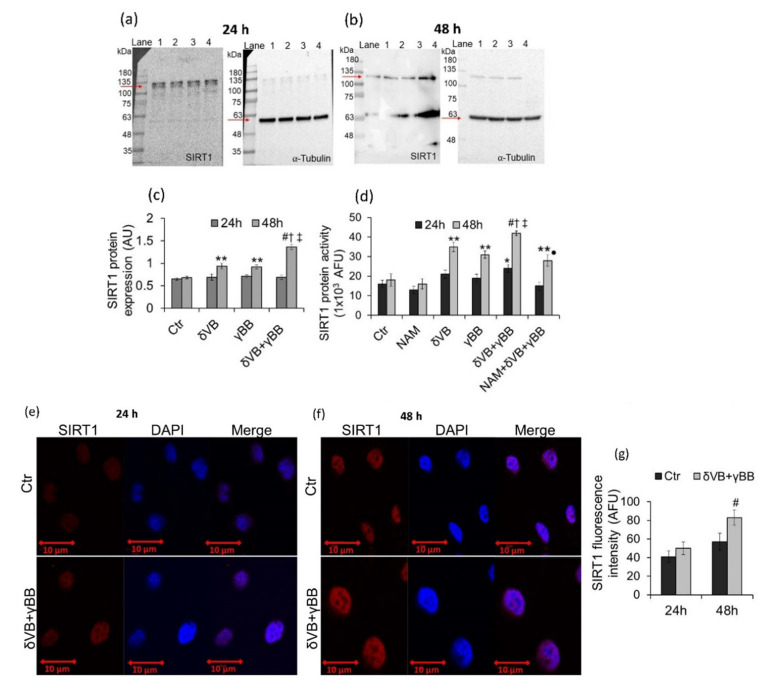
SIRT1 activation by δVB and γBB. (**a**–**c**) SIRT1 protein expression levels were determined in Cal 27 cells after exposure to δVB (2 mM), γBB (2.5 mM), or δVB + γBB (2 mM + 1.62 mM). Results were expressed as arbitrary units (AU) with ** *p* < 0.01 vs. Ctr, *^#^ p* < 0.001 vs. Ctr, ^†^ *p* < 0.05 vs. δVB, ^‡^ *p* < 0.05 vs. γBB. Lane 1 = Ctr, lane 2 = δVB, lane 3 = γBB, lane 4 = δVB + γBB. Arrows indicate SIRT1 protein bands at about 110-kDa. Nonspecific bands were detected at lower molecular weight. (**d**) SIRT1 enzyme activity expressed as arbitrary fluorescence units (AFU); * *p* < 0.05 vs. Ctr, ** *p* < 0.01 vs. Ctr, ^†^ *p* < 0.05 vs. δVB, ^‡^ *p* < 0.05 vs. γBB, ^#^ *p* < 0.001 vs. Ctr, • *p* < 0.01 vs. δVB + γBB (**e**,**f**) Representative confocal images of SIRT1 expression (red) in control (Ctr) cells and cells exposed to δVB + γBB. Nuclei were counterstained with DAPI (blue). Scale bar = 10 μm. (g) Fluorescence intensity values of SIRT1 was expressed as AFU with ^#^ *p* < 0.001 vs. Ctr.

δVB and γBB induced a positive regulation of SIRT1 protein expression levels in Cal 27 cells only at 48 h (*p* < 0.01 vs. Ctr), with no effects observed at 24 h (Figure 5a–c). Noteworthy, SIRT1 upregulation was more pronounced following treatment with δVB + γBB (*p* < 0.001 vs. Ctr). Results were confirmed by measurements of SIRT1 enzyme activity (*p* < 0.001 vs. Ctr, *p* < 0.05 vs. δVB, *p* < 0.05 vs. γBB) (Figure 5d). Moreover, δVB + γBB-induced SIRT1 enzyme activity was reduced by cell pre-treatment with SIRT1 inhibitor, nicotinamide (NAM) (*p* < 0.01 vs. δVB + γBB). Upregulation of SIRT1 protein expression in Cal 27 cells treated with δVB + γBB was also confirmed by confocal laser scanning microscopy analyses (Figure 5e–g).

### 2.6. ROS Levels

**Figure 6:**
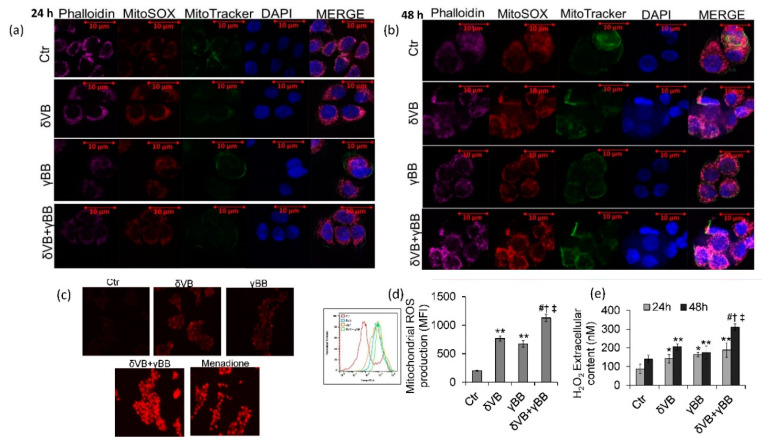
Mitochondrial stress assessment. (**a**,**b**). Representative images of confocal laser scanning analyses of Cal 27 mitochondrial ROS generation detected by MitoSOX and MitoTracker. Scale bars = 10 µm. Mitochondrial superoxide levels assessed by MitoSOX-based (**c**) fluorescence microscopy and (**d**) flow cytometry. Cells were treated with vehicle (Ctr), δVB (2 mM), γBB (2.5 mM), or δVB + γBB (2 mM + 1.62 mM) for 48 h in serum-free media. Results are expressed as median fluorescence intensity (MFI). (**e**) Extracellular H_2_O_2_ production was detected using the Amplex Red H_2_O_2_/peroxidase assay. Data are the mean±SD of three independent experiments with * *p* < 0.05 vs. Ctr, ** *p* < 0.01 vs. Ctr, *^#^ p* < 0.001 vs. Ctr, ^†^ *p* < 0.05 vs. δVB, ^‡^ *p* < 0.05 vs. γBB.

In order to better investigate the cellular events occurring during betaine-induced cytotoxicity in Cal 27 cells, chemical probes MitoSox and MitoTracker were used to evaluate the mitochondrial redox status (Figure 6a–d). Results showed that mitochondrial ROS production was activated by δVB (3.8-fold change) and γBB (3.3-fold change) (*p* < 0.01 vs. Ctr) starting from 24 h and continuing up to 48 h. The increase of MitoSox fluorescence was higher in cells treated with δVB + γBB (5.2-fold) (*p* < 0.05 vs. δVB, *p* < 0.05 vs. γBB, *p* < 0.001 vs. Ctr). These effects were accompanied by an increased extracellular ROS content, with the highest effect at 48 h of incubation with δVB + γBB (Figure 6e). In HaCaT cells, mitochondrial ROS levels were not affected by treatments (Supplementary Figure S5).

### 2.7. SIRT1 Mediates Betaine Anticancer Activity

**Figure 7:**
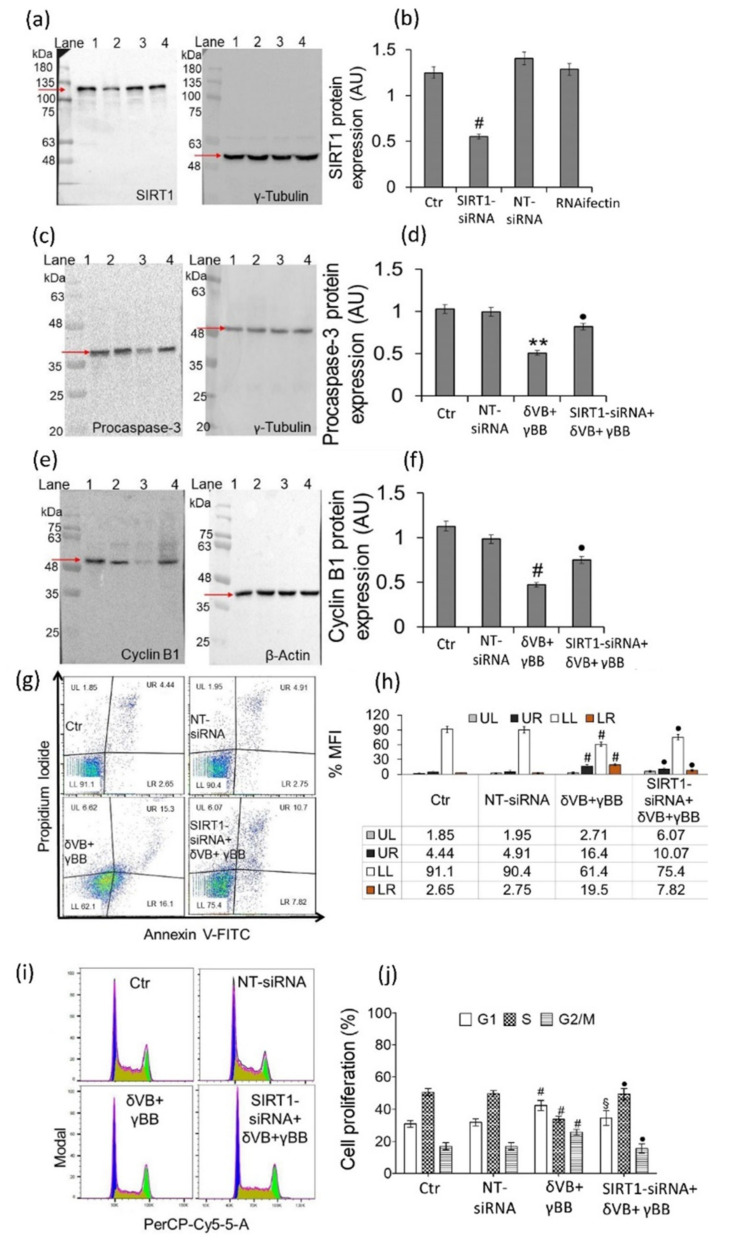
Cal 27 transient gene silencing of SIRT1. Cells were transfected with transfection reagent (RNAifectin), scramble siRNA (NT-siRNA), SIRT1-siRNA, or medium (Ctr). Treatments with betaines (48 h) were performed on transfected (SIRT1-siRNA + δVB + γBB) and non-transfected cells (δVB + γBB). After treatments, Western blots were performed to determine the expression levels of SIRT1, procaspase-3, and cyclin B1. (**a**,**b**) SIRT1 protein expression. Lane 1 = Ctr, lane 2 = SIRT1-siRNA, lane 3 = NT-siRNA, lane 4 = RNAifectin. Arrow indicates SIRT1 protein band at about 110-kDa. Nonspecific bands were detected at lower molecular weight. ^#^ *p* < 0.001 vs. Ctr. (**c**,**d**) Procaspase-3 protein levels. Lane 1 = Ctr, lane 2 = NT-siRNA, lane 3 = δVB + γBB, lane 4 = SIRT1-siRNA + δVB + γBB. ** *p* < 0.01 vs. Ctr; ^•^ *p* < 0.01 vs. δVB + γBB. (**e**,**f**) Cyclin B1 protein expression. Lane 1 = Ctr, lane 2 = NT-siRNA, lane 3 = δVB + γBB, lane 4 = SIRT1-siRNA + δVB + γBB. ^#^ *p* < 0.001 vs. Ctr, ^•^ *p* < 0.01 vs. δVB + γBB. (**g**,**h**) Representative dot plots of annexin V-FITC/PI-stained Cal 27 cells and percentage of apoptosis Quadrants show cell percentage; lower left (LL), viable cells; upper left (UL), necrotic cells; lower right (LR), early apoptotic cells; upper right (UR), late apoptotic cells. Fluorescent signal generated from each dye was analyzed by FlowJo software and results expressed as median fluorescence intensity (MFI). Data are expressed as mean ± SD of *n* = 4 experiments. At least 10,000 events were acquired. ^#^ *p* < 0.001 vs. Ctr, ^•^ *p* < 0.01 vs. δVB + γBB. (**i**,**j**) Representative cell cycle dot plots and analysis. Cell cycle distribution was assessed by flow cytometry collecting PI fluorescence as FL3-A (linear scale) and analyzed by ModFIT software (Verity Software House). ^#^ *p* < 0.001 vs. Ctr, ^•^ *p* < 0.01 vs. δVB + γBB, ^§^ *p* < 0.05 vs. δVB + γBB.

Molecular mechanism was investigated by transient SIRT1 gene silencing with small interfering RNA (SIRT1-siRNA) (Figure 7). Treatments with betaines were performed for 48 h on transfected (SIRT1-siRNA + δVB + γBB) and non-transfected cells (δVB + γBB). Results indicated that transfected cells, showing a marked decrease of SIRT1 expression (Figure 7a,b), responded to δVB + γBB (SIRT1-siRNA + δVB + γBB) with an upregulation of procaspase-3 (*p* < 0.01 vs. δVB + γBB) (Figure 7c,d) and cyclin B1 protein expressions (*p* < 0.01 vs. δVB + γBB) (Figure 7e,f). Moreover, silenced cells treated with combined betaines (SIRT1-siRNA + δVB + γBB) showed an increase of viable cells (*p* < 0.01 vs. δVB + γBB) and a decrease of late (*p* < 0.01 vs. δVB + γBB) and early apoptosis (*p* < 0.01 vs. δVB + γBB) (Figure 7g,h). These results, together with the return to G2/M cell cycle phase near to control values (*p* < 0.01 vs. δVB + γBB) and the reduction of G1 phase depletion (*p* < 0.05 vs. δVB + γBB) (Figure 7i,j), suggest the role of SIRT1 in mediating cell cycle changes and apoptosis.

## 3. Discussion

**Figure 8:**
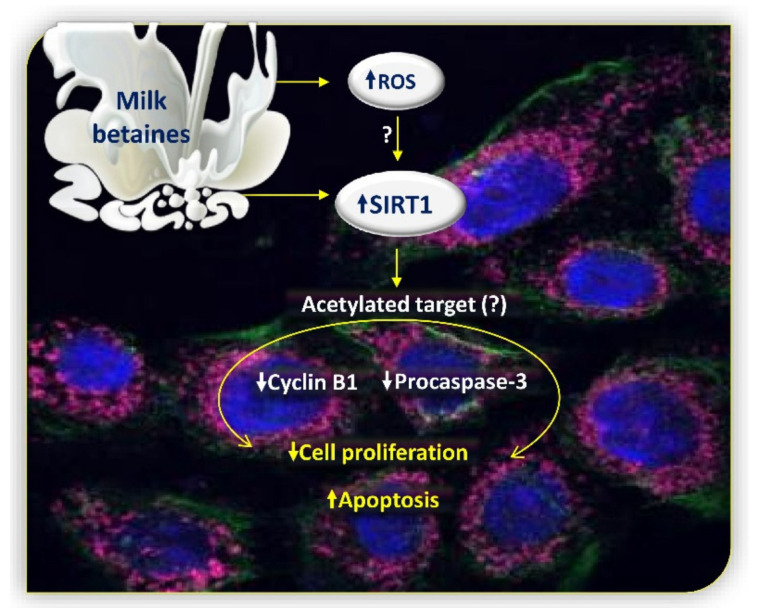
Schematic representation of cellular events induced by milk betaines in Cal 27 cells. Dietary betaines, δVB and γBB, cause inhibition of cell proliferation and induction of apoptosis. Pure betaines determine an increase in the accumulation of mitochondrial ROS which directly or indirectly influences the nuclear expression of SIRT1. Up-arrow, increase; down-arrow, decrease; question mark, unknown.

In the present study, we provide the first evidence that combined δVB and γBB, two betaines present in food from ruminant origin, display a synergistic action in inhibiting proliferation and inducing apoptosis in Cal 27 cells. Our data showed that combined betaines markedly increase mitochondrial ROS accumulation and SIRT1 deacetylating enzymatic activity in Cal 27 cells (Figure 8). Cell pre-treatment with SIRT1 inhibitor, nicotinamide, and SIRT1 gene silencing with small interfering RNA reduced betaine-triggered enzyme activity and apoptosis, respectively.

Cell proliferation is commonly impaired by apoptosis or cell cycle arrest [26]. The expression of cell cycle-related genes, such as cyclin A and cyclin B1, are necessary to trigger the G2/M transition. The downregulation of these genes, correlated with poorer outcomes in several cancer, suppresses the progression of cell cycle [27]. In this study, flow cytometry analysis revealed that combined betaines were more effective in inducing apoptosis and G2/M arrest in Cal 27 cells in time-dependent manner. Despite the expression of caspase-3, caspase-9 and PARP cleavage products were not appreciable, combined δVB and γBB downregulated procaspase-3, procaspase-9 and PARP expression levels, suggesting that Cal 27 cells undergo apoptosis. Moreover, the lack of cells in subG1 could be ascribable to DNA degradation not proceeding to internucleosomal regions but stopping after generating 50- to 300-kb fragments [28]. It also should be noted that if G2, M, or even late S-phase cells undergo apoptosis, the loss of DNA from these cells may not be adequate to place them at the sub-G1 peak, as they may end up with DNA content equivalent of that of G1 or early S-phase cells and, therefore, be indistinguishable from the latter [28].

Growing evidence supports the role of betaines as regulators of the cancer cell metabolism which exhibit increased aerobic glycolysis and oxidative stress associate with abnormal cell growth [29,30,31]. Inhibition of fatty acid oxidation results in an increased level of cytotoxic lipids, including proapoptotic ceramides containing palmitic and stearic acid which, in turn, rises the level of active caspase-3 and determines apoptosis [31]. Reprogrammed metabolic pathways in tumors facilitate the growth of malignant cells and support cell survival in the face of stressors present in the tumor microenvironment. It is conceivable that the effects observed in this study on the metabolic stress could be ascribed to changes in **l**-carnitine metabolism [32], not here approached, interfering with β-oxidation process and taking part to the stress induced cell death mechanism. This hypothesis is supported by the evidence that combined δVB and γBB are more active than single betaines in inducing mitochondrial damage, SIRT1 modulation, and apoptotic cell death. In colon cancer cells, ROS production induced by milk betaines was not a secondary effect but it triggered SIRT6 modulation and cell death, as demonstrated by reduced SIRT6 expression and pro-cell death effects when ROS generation was suppressed by the antioxidant N-acetyl-L-cysteine [8]. It is tempting to speculate that pure betaines cause an increased metabolic oxidative burden which might directly/indirectly affect nuclear expression of SIRT1. Nevertheless, it is evident that milk, even at µM concentration of betaines, displays a more robust effect on SIRT1 and cell proliferation compared to single tested betaines (mM), probably due to the occurrence of other known bioactive molecules, including short-chain acylcarnitines and small peptides [6,9].

Mechanistic studies, showed that δVB and γBB exert their function through the activation of SIRT1, strengthening its role as molecular target of these naturally occurring betaines. In particular, a combination of δVB and γBB showed highest potency in increasing SIRT1 protein expression and activity and its activation supports Cal 27 apoptosis and accumulation in the G2/M cell cycle phase, as demonstrated by SIRT1 gene silencing with small interfering RNA. A comprehensive understanding of cancer pathogenesis has been dedicated to the elucidation of the molecular mechanisms participating in multistep carcinogenesis and progression of oral squamous cell carcinoma. However, despite evidence for SIRT1 involvement in a variety of cell regulatory and physiological processes, the role of SIRT1 in regulating oral cancer progression and metastasis is poorly investigated. In Cal 27 cells, SIRT1 acts on specific targets, i.e., p53 and NF-κB, in mediating the anticancer effect of curcumin [22,33].

Age-related diseases, including cancer, result from one or more failures at cellular and molecular levels, making difficult the identification of molecular targets for their prevention and treatment. Cancer prevention is an important theme for healthy aging and the role of the quality of one’s diet for cancer prevention is definitely worthy of attention [34,35,36,37,38]. As for oral cancer, regarded as the main cause of death from oral diseases in many countries with the global estimates in 2018 of 354,864 new cases and 177,384 deaths, chemoprevention by dietary agents has evolved as a promising approach to control the incidence which has increased in many countries, especially in younger age groups [39,40]. The application of antioxidants or functional foods containing nutrients able to reverse or change epigenetic phenomena, makes nutrition a natural weapon to prevent cancer and improve health span [34,35,36,37,38]. In vitro evaluation of the anticancer effects of lactoferrin and tea polyphenol combination on Cal 27 cells showed a synergistic inhibition with induction of apoptosis and mitochondrial permeability transition [40]. Similarly, the in vivo and in vitro anticancer effects of curcumin in oral carcinomas is potentiated by combination with resveratrol [41,42].

Among dietary regulators of cellular redox homeostasis, δVB has been recently shown to induce a ROS-mediated apoptotic cell death in human colon cancer LoVo cells via SIRT6 activation and changes in mitochondrial integrity initiated by excessive ROS accumulation [8]. In this study, the cytotoxic effects of δVB in Cal 27 cells and its ability to positively modulate SIRT1 strengthen the observations about its potential role as epi-nutrient. Importantly, our data by demonstrating that δVB was more effective in combination with γBB, highlighted the multifaceted effects of the synergism between dietary compounds with health-promoting effects. Several bioactive food components are known to activate SIRT1, a nicotinamide adenine dinucleotide (NAD+)-dependent deacetylases (class III) involved in various cellular processes from aging to cancer [38]. It is undoubtedly hard to delineate the precise effect of a bioactive food component in modifying epigenetic phenomena, due to the simultaneous interaction with other nutrients, genes and lifestyle factors, adding complexity to the system. Although SIRT1 is involved in the anticancer activity exerted by combined betaines, nicotinamide did not completely return the treatment to control levels, suggesting that other independent mechanism(s) are likely involved. This hypothesis is also supported by the results showing that *SIRT1* silencing did not fully restore the levels of procaspase-3 and cyclin B1 expressions. However, the evidence here provided on the role of dietary δVB and γBB as novel nutritional epigenetic modulators underlines the importance of examining their potential on a broader panel of oral cancer cell lines and validating the in vivo efficacy.

## 4. Materials and Methods

### 4.1. Cell Culture and Treatments

Human tongue Cal 27 (CRL-2095) and pharynx FaDu (HTB-43) squamous carcinoma cell lines were obtained from ATCC (Manassas, VA, USA). Laryngeal UM-SCC-17A (SCC074) squamous carcinoma cells were from Sigma-Aldrich (St. Louis, MO, USA). Human keratinocytes HaCaT cells (300493) were from Cell Line Service (Eppelheim, Germany). Cal 27, UM-SCC-17A and HaCaT cells were cultured using Dulbecco’s Modified Eagle Medium (DMEM, Gibco, Life Technologies, Carlsbad, CA, USA, 21969035). FaDu cells were grown in Eagle’s Minimum Essential Medium (EMEM, Gibco, Life Technologies, Carlsbad, CA, USA, 670086). All cell culture media were supplemented with 100 U/mL penicillin, 100 mg/mL streptomycin (Gibco, Life Technologies, Carlsbad, CA, USA, 15070063) and 10% heat-inactivated fetal bovine serum (FBS, Gibco, Life Technologies, Carlsbad, CA, USA, 10270106). Cells were cultured at 37 °C in a fully humidified atmosphere of 5% CO_2_ and medium was changed every 3 days.

Cultured cells were treated with δVB, γBB (Sigma Aldrich, St. Louis, MO, USA, 403245) both dissolved in Hanks’ balanced salt solution (HBSS)-10 mM Hepes, or buffalo milk prepared as previously described [8]. δVB synthesis and purification was carried out as previously described [10]. Buffalo milk was collected from Italian Mediterranean buffaloes (*Bubalus bubalis*) bred in a commercial farm located in the South of Italy between 39.0° N and 41.5° N of Southern Italy. Animals were fed a total mixed ration consisting of maize silage, oat hay, green forage, corn meal and soybean meal and characterized by 0.91 milk forage units (MFUs), 15% crude protein on dry matter, and 18% starch, with a forage concentrate ratio of 70:30. Treatments were performed by culturing cells in complete medium with 3-kDa milk extracts, δVB, or γBB for a maximum time of 72 h. Control (Ctr) cells were treated with corresponding volumes of HBSS-10 mM Hepes. Milk extracts were prepared by centrifuging aliquots of bulk milk at 3000× *g* for 15 min at 4 °C to remove the fat globules. Skimmed milk was filtered through 5 μm Millipore filters. Aliquots were filtered through Amicon Ultra 0.5 mL centrifugal filters (3-kDa molecular weight cutoff). Before being used, milk extracts were filtered through 0.22 μm Millipore filters.

### 4.2. Cell Proliferation and Viability Assays

Cell proliferation was evaluated by seeding cells in 96-well plates at the density of 2 × 10^3^ cells/well in serum-free culture medium the day before treatments. As for cell viability, cells were seeded in 96-well plates at the density of 2 × 10^3^ cells/well in complete culture medium the day before treatments. Cell viability was expressed as number of viable cells following manufacturer’s instruction. Cells were treated with increasing volumes of milk (10, 15, 20 and 30% *v*/*v*) for different times. Betaine treatments were performed with increasing concentrations of δVB and γBB (up to 3 mM) in specific complete medium up to 72 h. To study the synergistic effects of δVB and γBB, oral cancer and non-tumor HaCaT cell lines were treated for 24, 48 and 72 h with δVB (2 mM) in the presence of increasing concentrations of γBB (up to 3 mM). Control (Ctr) cells were maintained in complete culture medium and treated with corresponding volumes of HBSS-10 mM Hepes. After treatments, cell proliferation and viability were detected using Cell Counting Kit-8 (CCK-8 Donjindo Molecular Technologies, Inc., Rockville, MD, USA, 20852) following manufacturer’s instruction. Briefly, 10 μL of CCK-8 solution was added to each well and cells were incubated at 37 °C for 4 h. Thereafter, absorbance was measured at 450 nm using a microplate reader model 680 Bio-Rad. All experiments were performed with *n* = 6 replicates. Cell proliferation and viability were expressed as the mean of the optical density at 450 nm ± Standard Deviation (SD) and as percentage of Ctr. Analyses were carried out using GraphPad Prism 6 software (GraphPad Software Company, San Diego, CA USA) while the Combination Index (CI) formulated by the CompuSyn 1.0 software (Paramus, NJ, USA).

### 4.3. Mitochondrial ROS Evaluation

To selectively measure the superoxide levels generated in the mitochondria of Cal 27 and HaCaT cells, Mitosox Red Mitochondrial Superoxide Indicator (Thermo Scientific, Rockford, IL, USA, M36008) was used following manufacturer’s protocol. Briefly, the levels of mitochondrial ROS were evaluated after treatment with δVB (2 mM), γBB (2.5 mM) or δVB (2 mM) *plus* γBB (1.62 mM) followed by staining with 5 μM Mitosox for 20 min at 37 °C. After washing with phosphate-buffered saline (PBS), cells were imaged on a fluorescence microscope EVOS FL Cell Imaging System (Thermo Scientific, Rockford, IL, USA). Then, Cal 27 and HaCaT cells were detached by trypsin/EDTA (Gibco, Life Technologies, Carlsbad, CA, USA, 25200056) and, after a PBS-washing, the median fluorescence intensity (MFI) was quantified using a BD Accuri™ C6. Data were analyzed by FlowJo V10 software (Vancouver, BC, USA). Mitochondrial ROS evaluation by Mitosox Red staining together with MitoTracker Green (Thermo Scientific, Rockford, IL, USA, M7514) labeling for mitochondria were also carried in 24 and 48 h-treated Cal 27 cells by confocal laser microscopy detection. Both vital dyes were performed on live cells, before the paraformaldehyde fixing, as reported in the “Confocal laser scanning microscopy” section.

### 4.4. Measurement of Extracellular H_2_O_2_ Using Amplex Red

The Amplex Red Hydrogen Peroxide/Peroxidase Assay Kit (Thermo Fisher Scientific, Waltham, MA, USA, A22188) was used to detect extracellular H_2_O_2_ released from cells after treatment with δVB, γBB, or δVB (2 mM) *plus* γBB (1.62 mM). After treatments, Cal 27 cells were trypsinized, dispersed thoroughly and counted to produce a 20 µL cell suspension containing 2 × 10^4^ live cells in Krebs-Ringer phosphate glucose buffer (145 mM NaCl, 5.7 mM sodium phosphate, 4.86 mM KCl, 0.54 mM CaCl_2_, 1.22 mM MgSO_4_, 5.5 mM glucose, pH 7.35). Cells were mixed with 100 µL Amplex Red reagent containing 50 µM Amplex Red and 0.1 U HRP/mL in Krebs-Ringer phosphate glucose buffer and incubated at 37 °C for 60 min. The fluorescence of the oxidized 10-acetyl-3,7-dihydroxyphenoxazine was measured at excitation wavelength of 530 nm and emission wavelength of 590 nm, using an Infinite 2000 Multiplate reader (Tecan, Männedorf, Swiss). H_2_O_2_ was quantified with an H_2_O_2_ standard curve within the concentration range of 0–2 µM.

### 4.5. Confocal Laser Scanning Microscopy

Cal 27 cells (10 × 10^3^/well) were seeded in 24-well plates containing microscope glass (12 mm) (Thermo Fisher Scientific, Waltham, MA, USA). After treatments, Mitosox Red, and MitoTracker Green staining, cells were fixed using 4% (*v*/*v*) paraformaldehyde solution for 20 min and then permeabilized with 0.1% (*v*/*v*) Triton X-100 in PBS for 10 min at RT. For SIRT1 immunofluorescence detection, the primary antibody (1:1000, Biorbyt, Cambridge, UK, orb306144) was incubated overnight at 4 °C, followed by incubation with Alexa Fluor 633 (1:1000, Life Technologies, Carlsbad, CA, USA) for 1 h. Phalloidin-iFluor 488 (1:1000, Abcam, Cambridge, UK, ab176753) was used to stain F-actin filaments. Nuclear staining was performed by using 2.5 µg/mL of 4′, 6-diamidino-2-phenylindole (DAPI, Sigma Aldrich, St. Louis, MO, USA) for 7 min. Microscopy analyses were performed using an LSM 700 confocal microscope (Zeiss, Oberkochen, Germany) with a plan apochromat X63 (NA1.4) oil immersion objective. The fluorescence intensity was evaluated with ImageJ 1.52n software (National Institutes of Health, Bethesda, MD, USA) and results expressed as arbitrary fluorescence units (AFU).

### 4.6. SIRT1 Enzyme Assay

Cal 27 cells were seeded in 96-well plates and treated with betaines. SIRT1 fluorometric drug discovery assay (Enzo Life, Farmingdale, NY, USA, BML-AK555-0001) was performed following manufacturer’s instruction. Briefly, 50 μL of fleur de lys substrate was added in each well and then plates were incubated for 5 h at 37 °C. In order to stop SIRT1 activity and to produce the fluorescent signal, 50 µL of Developer II was added. After incubation for 30 min at 37 °C, the fluorescence was measured at excitation wavelength of 360 nm and at emission light of 460 nm using a Tecan Infinite 2000 Multiplate reader.

### 4.7. Cell Cycle Distribution Analysis

Cal 27 and FaDu cells (3 × 10^4^/well) were seeded in 6-well plates and treated with δVB (2 mM), γBB (2.5 mM), or with δVB (2 mM) *plus* γBB (1.62 mM) up to 72 h. Cells were then detached with EDTA-trypsin, washed twice with PBS and stained 1 h in the dark in a propidium iodide (PI, Sigma Aldrich, St. Louis, MO, USA) solution (50 μg/mL PI, 25 μg/mL RNAse A, 0.1% sodium citrate, 0.1% triton in PBS). Flow cytometric analysis was performed using a BD Accuri™ C6 instrument (Becton Dickinson, San Jose, CA, USA) by collecting at least 10,000 events. Data analysis was performed with the ModFit LT V4.1.7 software.

### 4.8. Apoptotic Cells Analysis

In order to distinguish apoptotic (Annexin V-FITC-positive, PI-positive) from necrotic (Annexin V-FITC-negative, PI-positive) cells, the FITC Annexin V Apoptosis detection kit (BD Pharmigen, Franklin Lakes, NJ, USA, 556547) was used. As for cell cycle evaluation, Cal 27 and FaDu cells were treated with δVB (2 mM), γBB (2.5 mM), or δVB (2 mM) plus γBB (1.62 mM) up to 72 h. After trypsinization and 2-times PBS washing, cells were resuspended in 200 μL of Binding Buffer 1X and incubated with 2 μL Annexin V-FITC and 2 μL PI (20 μg/mL) for 30 min at room temperature. The detection of viable cells, early apoptotic cells, late apoptotic cells and necrotic cells was performed with a BD Accuri™ C6 system and data analyzed by the FlowJo V10 software. For each sample 20,000 events were recorded.

### 4.9. SIRT1 Gene Silencing

Cells were transfected with small interfering RNA (siRNA) (50 nM) and with control non targeting siRNA (NT-siRNA) (50 nM) using RNAifectin transfection reagent. SIRT1 pool siRNA consisted of a mixture of three sequences designed for specific Human Sirtuin 1 (Applied Biological Materials Inc. Richmond, BC, Canada). Transfection was performed following the manufacturer’s instructions. Briefly, Cal 27 cells (3 × 10^4^/well) were seeded in 6-well plates in complete culture media. Growth medium was removed after 24 h and transfection complexes (siRNA-RNAifectin) were added to serum-free and antibiotic-free medium. Cells were incubated for 16 h, followed by additional 24 h of incubation after the addition of FBS (10%) directly to each well. Treatments with betaines were performed for 48 h on transfected (SIRT1-siRNA + δVB + γBB) and non-transfected cells (δVB + γBB). After treatments, SIRT1, procaspase-3 and cyclin B1 expression levels, cell cycle, and apoptosis were monitored.

### 4.10. Preparation of Cell Lysates and Western Blotting Analysis

Cal 27 cells were lysed in RIPA lysis buffer (1% NP-40, 0.5% sodium deoxycholate, 0.1% SDS in PBS) containing 10 μg/mL aprotinin, leupeptin and 1 mM phenylmethylsulfonyl fluoride (PMSF). Cell lysates were incubated on ice for 1 h and centrifuged at 10,000× *g* for 15 min at 4 °C. Supernatants were recovered and protein content was determined by Bio-Rad Protein Assay kit (Bio-Rad, Hercules, CA, USA) and compared with a BSA standard curve. Total protein extracts (20–50 µg) were subjected to sodium dodecyl sulfate-polyacrylamide gel electrophoresis (SDS-PAGE) and transferred to nitrocellulose membranes (Bio-Rad). Opti-Protein XL Marker prestained protein ladder molecular weight markers used were from Abm (Applied Biological Materials Inc. Richmond, BC, Canada, G266). Membranes were blocked in 10 mM Tris-HCl, pH 8.0, 150 mM NaCl, 0.05% Tween 20 (TBST) supplemented with 5% nonfat dry milk for 1 h at room temperature. Membranes were incubated overnight at 4 °C with specific primary antibodies anti-SIRT1 (1:1000, orb306144), anti-SIRT6 (1:1000, ab62738), anti- poly(ADP ribose) polymerase (PARP, 1:1000, Cell Signaling Technology, Danvers, MA, USA, 9532), anti-caspase-3 (1:1000, Cell Signaling Technology, 9662), anti-caspase-9 (1:1000, Cell Signaling Technology, 9508), anti-cyclin A2 (1:1000, Cell Signaling Technology, 4656), anti-cyclin B1 (1:1000, Cell Signaling Technology, 4138), anti-α-tubulin (1:5000, Cell Signaling Technology, 3873), anti- γ-tubulin (1:5000, Sigma Aldrich, St. Louis, MO, USA, GTU-88), and anti-β-actin (1:5000, Cell Signaling Technology, 3700). After 1 h incubation with HRP-conjugated secondary antibodies (ImmunoReagents Inc. Raleigh, NC, USA GxMu-003-DHRPX and GtxRb-003-DHRPX), blots were examined by ECL detection Kit (Immobilon Western Chemiluminescent HRP Substrate, Millipore, Burlington, MA, USA, WBKLS0100) and analyzed by using Image Lab 5.2.1, Molecular ImagerChemiDoc XRS Imaging system (Bio-Rad Laboratories, Milan, Italy). After background subtraction, the densities of immunoreactive bands were measured with the ImageJ 1.52n software (National Institutes of Health). Loading control (α-tubulin, γ-tubulin, and β-actin) were used for protein expression normalization and results expressed as arbitrary units (AU).

### 4.11. Statistical Analysis

All reported results refer to experiments performed at least three times. Data are expressed as mean ± standard deviation (SD). Statistical differences were assessed by One-way ANOVA followed by Bonferroni’s post-hoc tests. *p* values < 0.05 were considered significant.

## 5. Conclusions

Our study showed new evidence on the synergistic anticancer effect of δVB and γBB, two dietary betaines naturally occurring in meat, milk and dairy products. These results unveil the potential of these two betaines in reducing the risk of tongue squamous cell carcinoma and pave the way for further research on innovative strategies to optimize their content in food to sustain human health and wellbeing.

## Supplementary Materials

The following are available online at https://www.mdpi.com/2072-6694/12/9/2468/s1, Figure S1: Milk effect on oral cancer cell viability and proliferation; Figure S2: Inhibition of UM-SCC-17A cell proliferation; Figure S3: FaDu cell cycle analysis; Figure S4: FaDu apoptotic cell death; Figure S5: Mitochondrial stress assessment in non-tumor cell line.
