# Supplementary material for: Synergistic Effect of Dietary Betaines on SIRT1-Mediated Apoptosis in Human Oral Squamous Cell Carcinoma Cal 27
Source: Cancers (Basel). 2020 Aug 31;12(9):2468. doi: 10.3390/cancers12092468 (PMC7563158; doi:10.3390/cancers12092468)
Supplement: Supplementary file 1 [file cancers-12-02468-s001.pdf]

*Type of the Paper (Article)*

# Synergistic Effect of Dietary Betaines on SIRT1-Mediated Apoptosis in Human Oral Squamous Cell Carcinoma Cal 27

**Nunzia D'Onofrio[1], Luigi Mele[2], Elisa Martino[1], Angela Salzano[3], Brunella Restucci[3], Domenico Cautela[4], Marco Tatullo[5], Maria Luisa Balestrieri[1*], Giuseppe Campanile[3]**

## Supplementary Figures

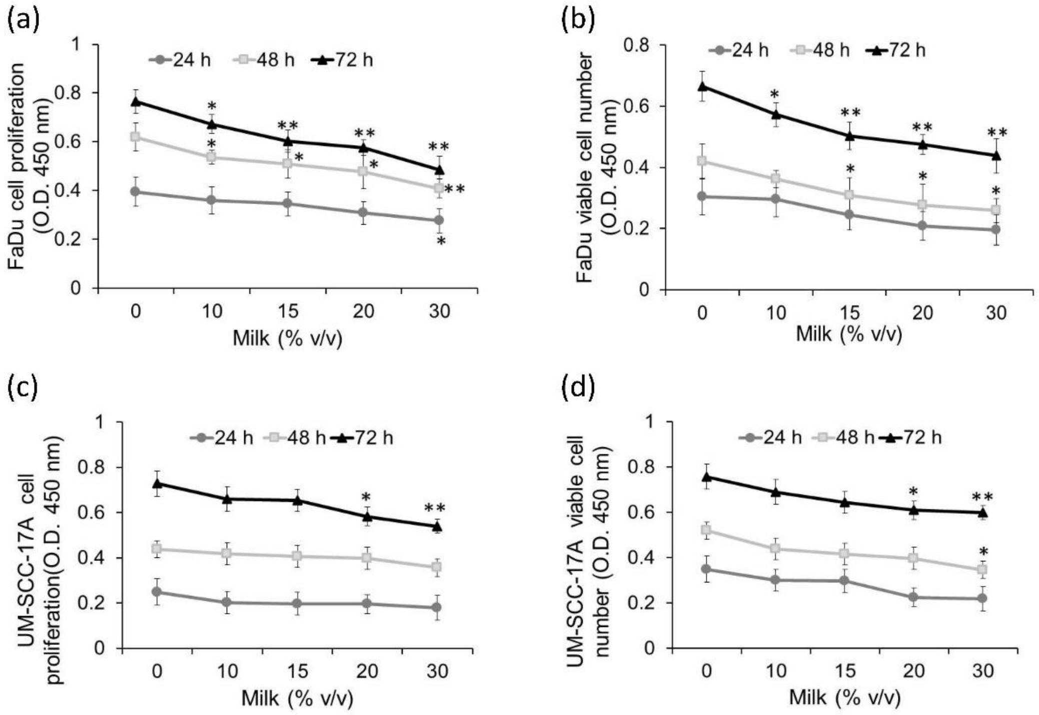


**Supplementary Fig.1 Milk effect on oral cancer cell viability and proliferation.** Cell viability and proliferation assessed by Cell Counting Kit-8 in (**a, b**) FaDu and (**c, d**) UM-SCC-17A cells treated for 24, 48 and 72 h with increasing volumes of milk (up to 30% v/v). Milk was centrifuged at 3,000 x g for 15 min at 4°C to remove fat globules. Skimmed milk was then filtered through a 5 µm Millipore filter followed by filtration through an Amicon Ultra 0.5 mL centrifugal filter with a 3-kDa molecular weight cut-off. Before being used, milk extracts were filtered through 0.22 µm Millipore filters. Values represent the mean±SD of three independent experiments. **P*<0.05 *vs* Ctr, ***P*<0.01 *vs* Ctr.

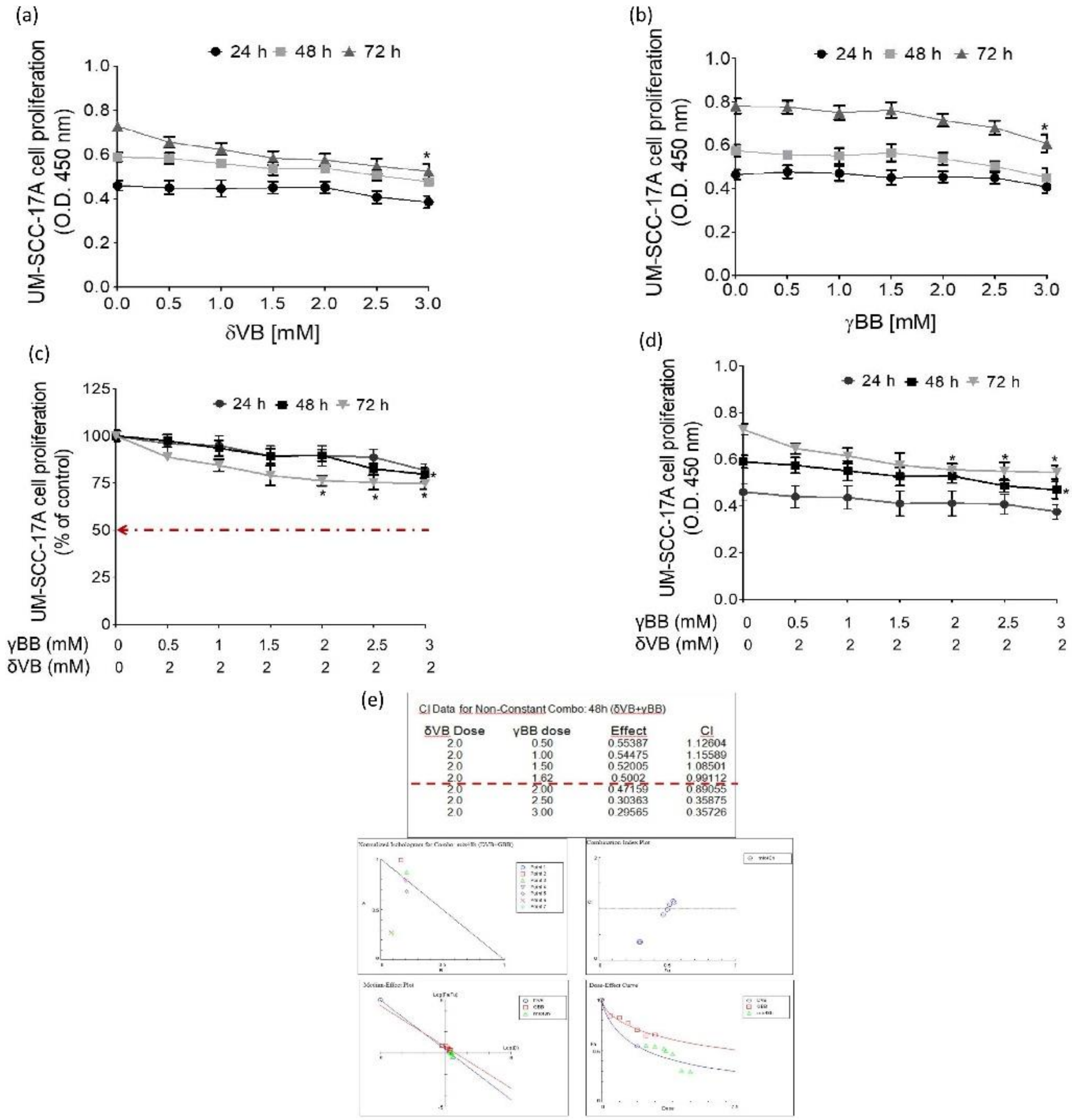


**Supplementary Fig.2. Inhibition of UM-SCC-17A cell proliferation**. Different concentrations (up to 3 mM) of δVB or γBB were used to treat (**a, b**) UM-SCC-17A cells for 24, 48 and 72h. (**c, d**) Cell proliferation was assessed after treatment with δVB (2 mM) plus serial concentrations of γBB (0.5, 1, 1.5, 2, 2.5, 3 mM). Control cells were grown in medium containing the same volume (% v/v) of HBSS-10 mM Hepes. Cell proliferation inhibition was assessed using Cell Counting Kit-8 assay. (**e**) Combination index values and dot plots resulted by using serial concentrations of γBB at δVB (2 mM). Values represent the mean±SD of four independent experiments. *$P$<0.05 *vs* Ctr.

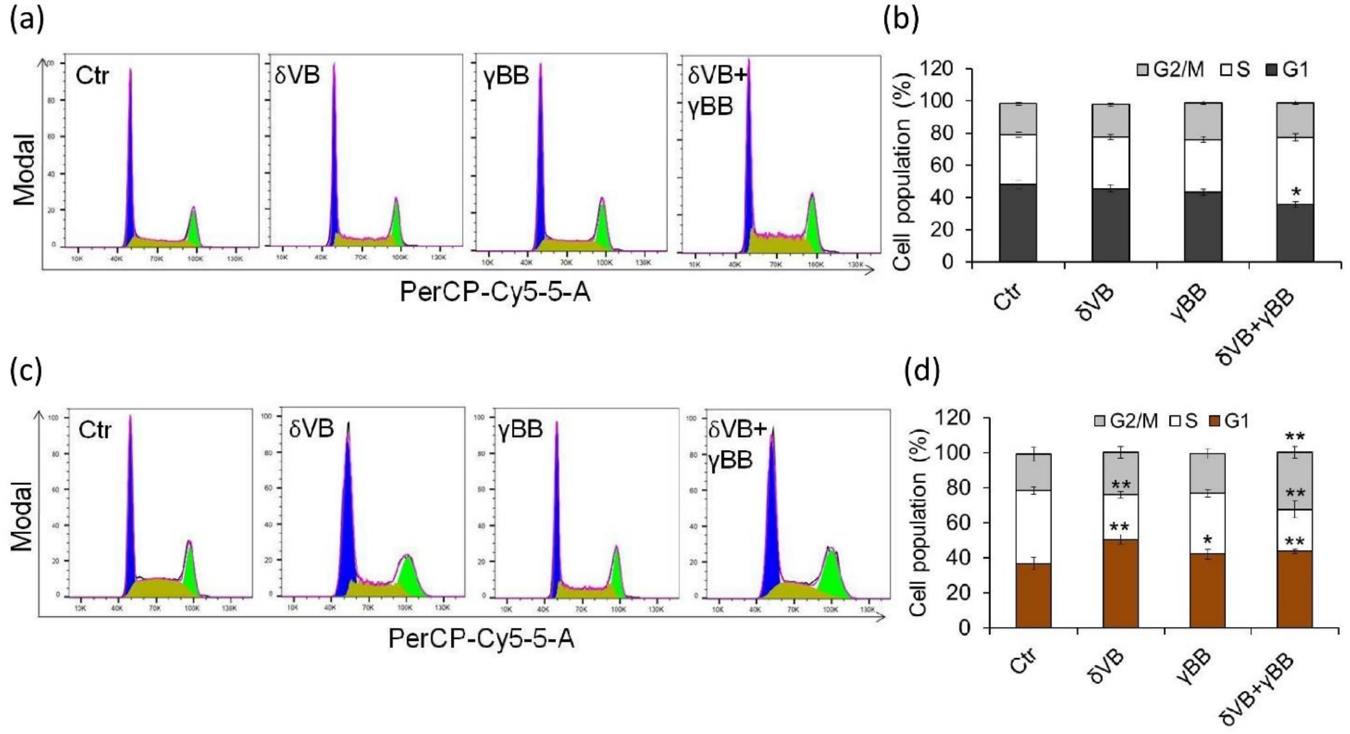


**Supplementary Fig.3. FaDu cell cycle analysis**. Cells were treated with vehicle (Ctr), δVB (2 mM), γBB (2.5mM) or δVB+γBB for (**a, b**) 48 and (**c, d**) 72 h. Cell cycle distribution was assessed by flow cytometry collecting PI fluorescence as FL3-A (linear scale) and analysis by ModFIT software (Verity Software House, USA, Becton Dickinson). For each sample at least 10.000 events were analyzed. *$P$<0.05 *vs* Ctr, **$P$<0.01 *vs* Ctr.

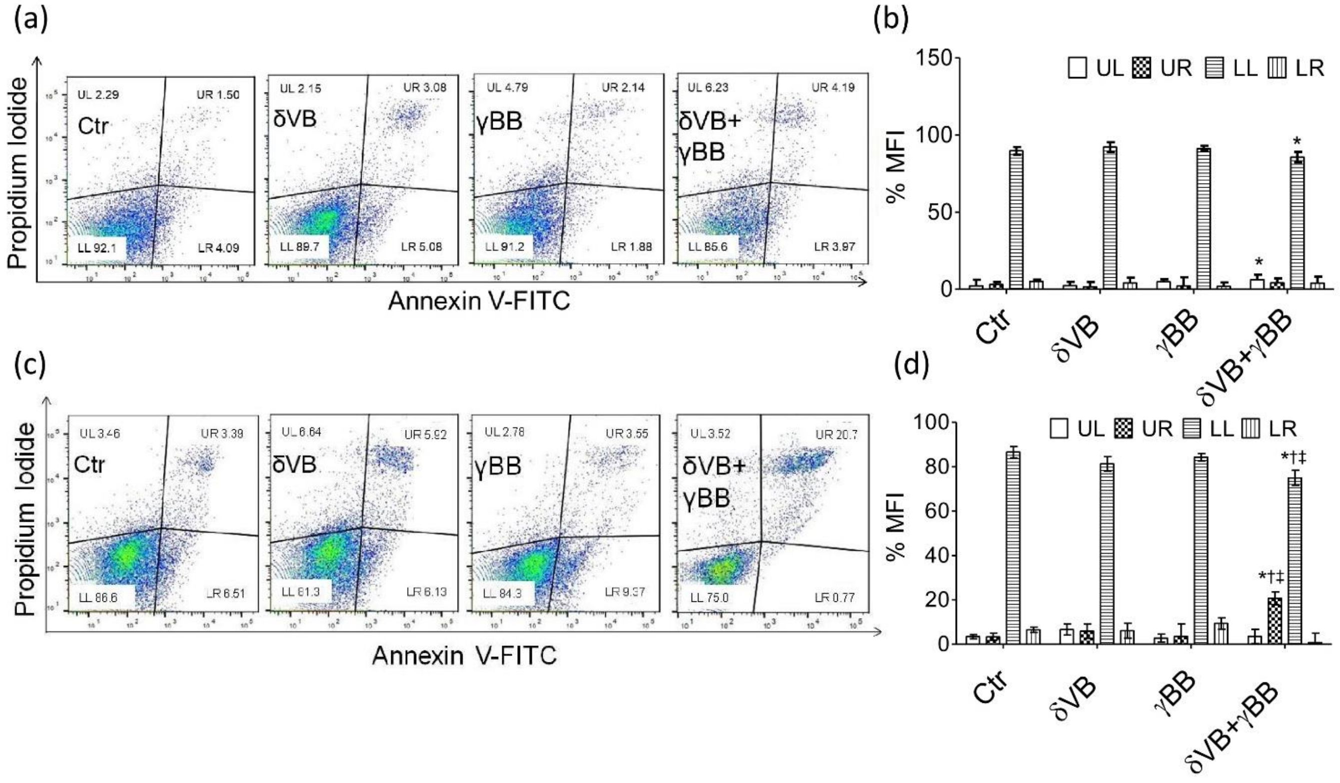


**Supplementary Fig. 4. FaDu apoptotic cell death.** Representative dot plots and analysis of annexin V-FITC and PI-stained cells analyzed after (**a, b**) 48 and (**c, d**) 72 h of treatment by flow cytometry. Data are expressed as mean±SD of n=3 experiments. At least 10.000 events were acquired. $^{*}P<0.05$ *vs* Ctr, $^{\dagger}P<0.05$ *vs* δVB, $^{\ddagger}P<0.05$ *vs* γBB.

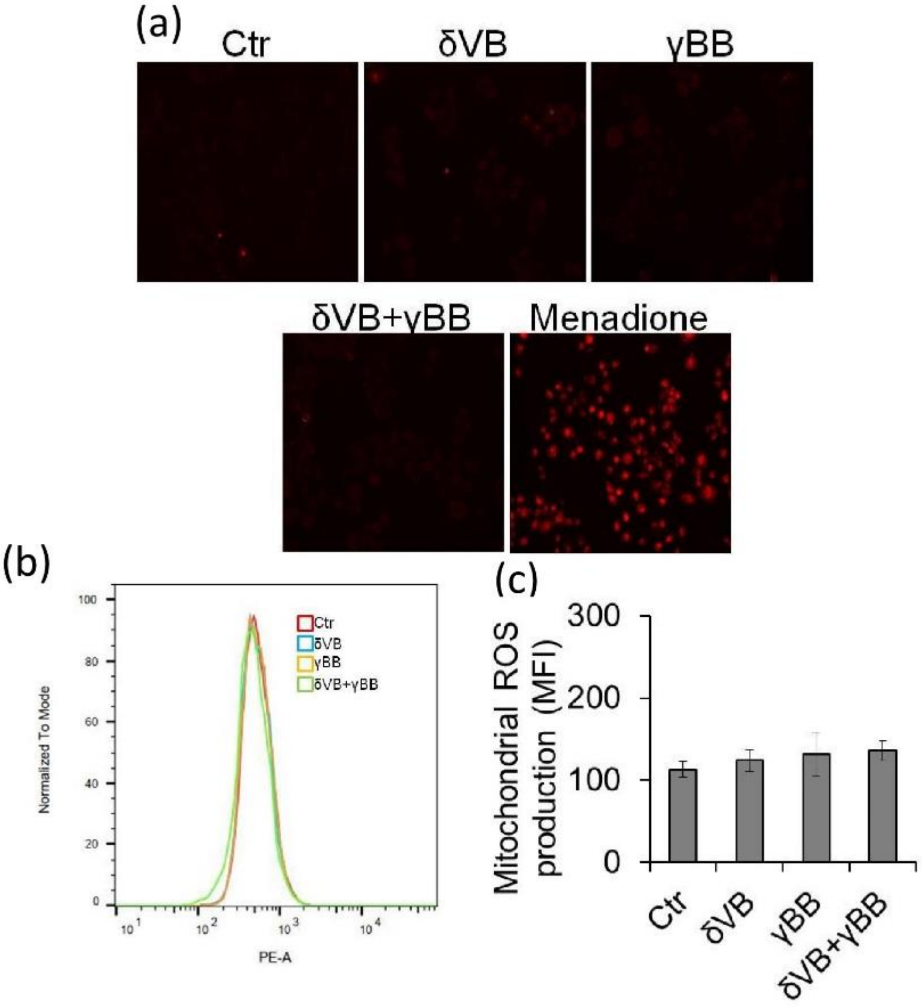


**Supplementary Fig. 5. Mitochondrial stress assessment in non-tumor cell line**. HaCaT cells were treated with vehicle (Ctr), δVB (2 mM), γBB(2.5mM) or δVB+γBB for 72 h in serum-free media. After MitoSOX staining, cells were analyzed by (**a**) fluorescence microscopy and (**b, c**) FACS analysis. Menadione is used as positive control.
